# Environmental Co-Exposure to Lead and Manganese and Intellectual Deficit in School-Aged Children

**DOI:** 10.3390/ijerph15112418

**Published:** 2018-10-31

**Authors:** José A. Menezes-Filho, Chrissie F. Carvalho, Juliana L. G. Rodrigues, Cecília F. S. Araújo, Nathália R. dos Santos, Cássio S. Lima, Matheus J. Bandeira, Breno L. de S. Marques, Ana Laura S. Anjos, Homegnon A. F. Bah, Neander Abreu, Alline Philibert, Donna Mergler

**Affiliations:** 1 Laboratory of Toxicology, School of Pharmacy, Federal University of Bahia, Salvador 40170-115, Brazil; julgrodrigues3@gmail.com (J.L.G.R.); nathalias@ufba.br (N.R.d.S.); matheus.jesus@ufba.br (M.J.B.); anjos.ana@ufba.br (A.L.S.A.); ferreol40@hotmail.com (H.A.F.B.); 2 Institute of Psychology, Federal University of Bahia, Salvador 40170-115, Brazil; chrissie.carvalho@pro.ucsal.br (C.F.C.); cassio.lima@ufba.br (C.S.L.); lsmbrenoa@gmail.com (B.L.d.S.M.); neander.abreu@ufba.br (N.A.); 3 Environmental and Public Health Program, National School of Public Health, Oswald Cruz Foundation, Rio de Janeiro 21041-210, Brazil; cecilia.araujo@posgrad.ensp.fiocruz.br; 4 Centre de Recherche Interdisciplinaire sur la Biologie, la Santé, la Société et l’Environnement (CINBIOSE), Université du Québec a Montreal, Montreal, QC H3C 3P8, Canada; philibert.aline@uqam.ca (A.P.); mergler.donna@uqam.ca (D.M.)

**Keywords:** lead, manganese, children, intellectual function, environmental contamination

## Abstract

Studies have demonstrated that, for urban children, dust represents the main exposure to sources of metals like lead (Pb) and manganese (Mn). We aimed to investigate the exposure to these metals and their association with intellectual deficit in children living in an industrial region. This cross-sectional study recruited volunteers from four elementary schools in the town of Simões Filho, Brazil. We evaluated 225 school-aged children (7–12 years) for blood lead (PbB) and manganese hair (MnH) and toenails (MnTn) by graphite furnace atomic absorption spectrometry. Child and maternal IQs were estimated using the Wechsler Abbreviated Scale for Intelligence (WASI). Median and range PbB were 1.2 (0.3–15.6) μg/dL. MnH and MnTn medians (ranges) were 0.74 (0.16–8.79) μg/g and 0.85 (0.15–13.30) μg/g, respectively. After adjusting for maternal IQ, age and Mn exposure, child IQ drops by 8.6 points for a 10-fold increase in PbB levels. Moreover, an effect modification of Mn co-exposure was observed. In children with low MnTn, association between Pb and child IQ was not significant (β = −6.780, *p* = 0.172). However, in those with high MnTn, the association was increased by 27.9% (β = −8.70, *p* = 0.036). Low Pb exposure is associated with intellectual deficit in children, especially in those with high MnTn.

## 1. Introduction

The World Health Organization estimates that 15–18 million children in developing countries suffer from permanent brain damage due to lead (Pb) poisoning [1]. Historically, leaded gasoline has been responsible for more than 90% of human Pb exposure. However, the global elimination of leaded gasoline over the past 25 years has resulted in important benefits, with more than 1.2 million premature deaths avoided each year (125,000 among children). It has been estimated that the annual cost of childhood Pb exposure in the United States is $50 billion [2] and for every $1 invested to reduce Pb hazards in housing units, society would benefit by an estimated $17 to $221, a cost–benefit ratio similar to the ratio for childhood vaccines [3]. A population survey administered by the Centers for Disease Control and Prevention (CDC) showed that the geometric mean for blood lead (PbB) levels for American children 1–5 years of age fell from 14.9 µg/dL in 1976 to 1.7 µg/dL in 2006 [4], similar to levels observed in adults from the National Health and Nutrition Examination Survey (NHANES) 2007–2010, which showed to increase along the lifespan [5]. Brazil achieved a total phase-out of leaded gasoline in 1991, and PbB might have dropped since then; however, there is no population-based screening of children’s PbB in Brazil to confirm that potential decrease.

Lead is a ubiquitous metal that can be found in different source materials and products, including paint pigments, soil, drinking water, food, cosmetics, gasoline, herbal products, and ceramics [6,7]. Human exposure occurs mainly through the respiratory and gastrointestinal tracts, as well as dermal contact. As children are known for their “hand to mouth” behavior, especially for ages one to five years, the oral route is of more importance for them where dust exposure represents an important source of Pb [8]. Respiratory tract is another frequent route of exposure for children, who, as they tend to spend more time outdoors, may absorb more airborne metals since their respiration and metabolic rates are higher than adults [9].

Social vulnerability is another important risk factor for Pb exposure [10,11]. Children may be more exposed to Pb from the environment if they live near major roads with high traffic intensity and near domestic or industrial waste incinerators or live with smokers [12]. Menezes-Filho et al. [13] showed that children living with parents who burned household wastes had significantly higher PbB and Pb in the hair than children whose parents did not burn waste. Exposure to Pb in children is associated with developmental problems, including impaired cognitive function, attention deficit, hyperactive behavior, hearing impairment, and reduced stature [14,15,16,17]. Cognitive activity and brain development occur simultaneously [18,19]. It is has been suggested that inorganic Pb ions can disrupt transmitter release at several sites critically involved in Ca^2+^-regulated secretion [20,21]. Karri et al. [20] in a review states that prenatal exposure to Pb influences the long-term potentiation machinery in the developing brain by disrupting the N-methyl-d-aspartate (NMDA) receptor expression in the hippocampus region that results in cognitive deficit in children.

Contrary to Pb, manganese (Mn) is an essential micronutrient, necessary for several vital functions, which at elevated concentrations can accumulate in the brain and induce neurotoxicity [22,23]. The biological mechanisms by which Mn exerts toxicity in humans have not yet been fully elucidated. It is known that excessive Mn absorption can cause its accumulation, mainly in the basal ganglia with consequent neurodegeneration of this region [24,25,26]. Indeed, there is also strong evidence that Mn can also produce chemical and structural changes in the brain’s cortical regions affecting cognitive and memory functions [22,27].

Very few studies have looked at the effect of the co-exposure to these metals on cognitive function. Lucchini et al. [28], in a cohort of Italian adolescents, observed a decrease in total IQ score with increasing PbB levels and no effect of Mn exposure on IQ nor any interaction with Pb was observed. On the other hand, Pb’s effect on psychomotor development scores was more pronounced in children with higher exposure to Mn in a Mexican cohort [29], in Bangladeshi children [30,31], and a similar interaction was observed in Taiwan birth cohort study [32].

Previously, we have demonstrated that children who attended elementary schools with high Mn dust deposition rates due to the emissions from the ferro-manganese alloy plant presented higher levels of Mn measured in hair and toenails [33]. However, Pb dust deposition rates were not related with the proximity to that plant, but more associated with the vicinity of the schools to high traffic roads. The objectives of this current study are: (i) to investigate if the observed PbB levels in those children are associated with intellectual deficits; (ii) and to test whether there would be an effect of the co-exposure to Mn on child IQ scores.

## 2. Materials and Methods

### 2.1. Study Design and Population

**Figure 1:**
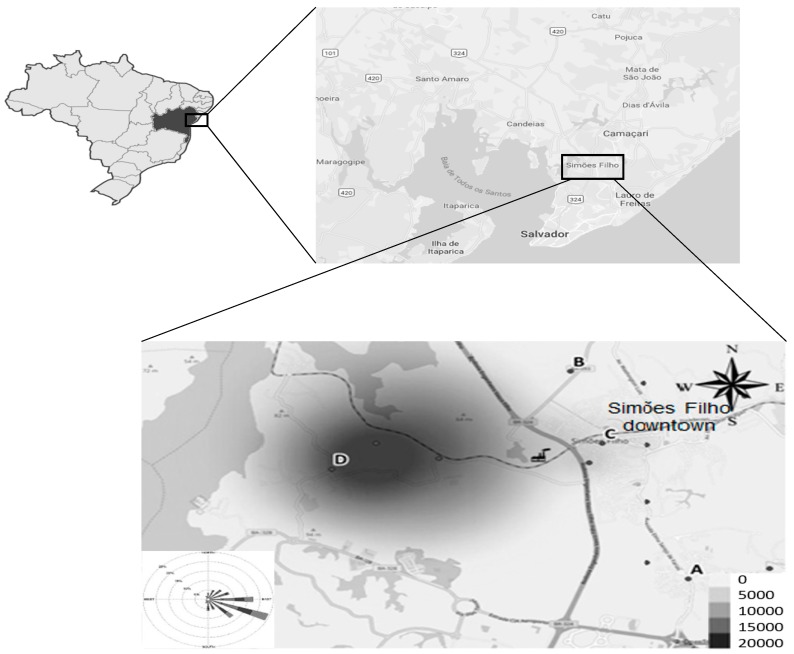
Schematic map of the study area, showing the location of the Mn alloy plant and the four elementary schools (A–D) attended by the study population.

A cross-sectional study was carried out in the municipality of Simões Filho, located in the metropolitan region of Salvador, Bahia, Brazil. The town, which is located on the edge of the main highway reaching the state capital, is exposed to diverse atmospheric pollution emitted by cement, petrochemical, and shipyard related activities and particularly affected by the ferro-manganese alloy plant [34], located approximately 2 km from the urban center (Figure 1). Four elementary schools were selected in this municipality, based on their distance to the alloy plant and the levels of Mn deposition rate in dust reported in our previous publication [34]. Children’s inclusion criteria were: living in the region for at least one year, aged 7 to 12 years, and attending one of the selected schools. The non-inclusion criteria were history of neurological disorders or psychiatric follow-up. Recruitment was done in the selected schools, after the principal provided us with the lists of registered students in each school level. We sent out invitations to parents or caregivers inviting them to attend meetings, where we explained the objectives and study procedures. The parents or caregivers signed a free and informed consent form authorizing the participation of their children in the investigation. The children also provided a written consent agreeing to participate as volunteers. This study protocol was approved by the Federal University of Bahia Research Ethics Committee (No. 874.463/2014).

### 2.2. Questionnaires and Anthropometric Measurements

Demographic, socio-economic characteristics, and lifestyle and health habits (exposure to cigarette smoke, nutrition status, etc.) data were obtained using semi-structured questionnaires by interview to the parents or primary caregivers. The socio-economic classification of the study population uses five descending classes (A to E), based on possession of comfort items at home, according to the Brazilian association of Population Studies [35]. Weight measurements were performed on an upright scale (UP15OS Urano^®^) with a capacity of 150 kg. Height was measured with the aid of a portable stadiometer (Welmy^®^) with 0.5 cm intervals attached to the wall. Body mass index (BMI) was calculated by dividing the weight in kilograms by the square of the height in meters. The chronic nutritional index, height-for-age (HA) z-score, encompasses growth and stature, which is inversely related to protein, calcium, and iron deficiency during early childhood. It was calculated using the AnthroPlus software, based on the WHO reference population collected in 2007 for people aged 5–19 years [36].

### 2.3. Exposure Biomarkers

Blood samples were collected by venous-puncture in the cubital portion into vacuum tubes suitable for trace metals (Vacutainer^®^ with blue cap BD). Since Pb affects the heme synthesis and Mn absorption is increased in iron-deficient children, hemoglobin (Hb) and iron (Fe) levels were also determined in blood samples. Blood lead (PbB) levels were analyzed by graphite furnace atomic absorption spectrometry (GFAAS) on the AA240Z, GTA 120 equipment (Varian, Palo Alto, CA, USA), following the methodology described by Menezes-Filho et al. [12]. For quality assurance purposes, blood samples of the Blood Lead Analysis Proficiency Program of the Adolfo Lutz Institute (Brazil) were used. The method presented accuracy of 97.5% and precision of 2.9%. The limit of detection (LOD) was set at 0.3 μg/dL and the results below this limit expressed as LOD/2.

Hair and toenail sample collection and analysis are described in detail in our previous report [33]. Briefly, hair was cut as close to the scalp from the occipital region and all toenails were clipped from both feet using a stainless-steel nail clipper. Hair and toenail samples were cleaned for external contamination and mineralized by microwave assisted digestion. The mineralized samples, reagent blanks, and reference material for quality assurance purpose (Human hair IAEA-085, International Atomic Energy Agency, Vienna, Austria) were analyzed in duplicates by GFAAS on the same equipment. Accuracy and precision observed were 101% and 1.4%, respectively. The procedural limit of detection (LOD) established was 0.1 μg/g and no sample result was below this limit.

### 2.4. Neuropsychological Assessment

All neuropsychological evaluations were carried out individually in a quiet room at each school in the same period as the collection of the biological samples and application of the questionnaires. Trained psychologist coordinated by the leading neuropsychologist (CFC) applied all test and were unaware of the child’s exposure status. The Wechsler Abbreviated Intelligence Scale (WASI) [37], a brief, internationally recognized tool available in Brazil since 2014 for people aged 6 to 89 years, was used to evaluate child and maternal intellectual performance. For obtaining the estimated IQ, the sub-tests Vocabulary and Matrix Reasoning were used. The Vocabulary subtest evaluates semantic knowledge and the Matrix Ratio subtest evaluates nonverbal logical reasoning ability, a task that evaluates fluid intelligence. Maternal intelligence was also evaluated at the schools attended by the children upon scheduled invitation at the same time of questionnaire application.

### 2.5. Statistical Analysis

From the 225 children who participated in the study, a total of 216 IQ data points was used for statistical analyses. A series of descriptive statistical analyses were carried out on biomarkers of exposure (MnH, MnTn, and PbB) and on children’s and maternal intellectual performance (IQ scores). To assess the strength of the association between biomarkers of exposure, IQ scores, and common epidemiological characteristics of the study population (health habits and demographics), initially, non-parametric Spearman correlation analyses were performed. Since the distributions of biomarker levels were skewed, data were log-10 transformed for further analyses.

A series of multivariate linear regression (MLR) models was performed to identify which biomarkers (PbB, MnH, or MnTn) were significantly associated with child IQ, using the enter approach. Two PbB observations identified as outliers were removed from the dataset (15.6 and 7.6 µg/dL) before running the models. Initially, we tested common covariables known to be associated with intellectual performance, as sex, age, nutritional status, and metal biomarkers identified in the bivariate analyses. Since, maternal IQ scores were not available for almost 40% of the children, we tested two models: without maternal IQ and then with maternal IQ. Afterwards, we tested for Mn exposure interaction with PbB levels, entering the interaction terms (LogPbB*LogMnH and LogPbB*LogMnTn) in the regression model. Finally, we tested for the effect modification of Mn exposure on the association of PbB on IQ, by comparing the LogPbB beta coefficients among children with low exposure to Mn (LowMnTn<Median) versus children with high Mn exposure (HighMnTn>Median). The level of statistical significance in the all statistical analyses was defined as *p* < 0.05. The analyses were performed using SPSS software version 22 for Windows.

## 3. Results

### 3.1. Population Characteristics

Table 1 provides a description of the study population characteristics and IQ scores. The mean age of the children was 116.8 months ranging from 84.0 to 156.9 months. The average BMI was 17.6 (SD 12.7–44.7) kg/m^2^, with few children classified as overweight (11.8%), obese (10.5%), and 1.4% classified with morbid obesity. For the 186 children for whom hemoglobin was measured, 5.5% were classified as anemic (Hb < 11.5 g/dL). The main caregivers were mothers (83.6%), followed by fathers (6.2%) and others (10.2%), including grandparents, aunts, and stepmothers. For the majority of children (75.2%), the gestational period took place in the study region. Maternal schooling was low, 76.0% did not attain high-school. The socioeconomic classification is considered low income (Classes C and D/E representing 97.6%), but not below the poverty line. As far as paternal occupation is concerned: The most frequent occupation was driver (10%), followed by working on civil construction (8%), and service as security (7.8%), and there were several other occupations reported but none in mining, welding or in chemical industry. Maternal occupation was limited: About half (52%) worked at home, service as household cleaning (11%), and seller (15%) (data not shown). The presence of at least one smoker at home was reported for 18.1% of the children.

### 3.2. Biomarkers of Exposure and Bivariate Analysis

The descriptive statistics of metal biomarkers are presented in Table 2. PbB distribution was skewed to the right with a median of 1.15 (range 0.2–15.6) µg/dL. Only 5 children had PbB equal or below the LOD (2.5%) and 98.2% of the children presented PbB equal or below 5 µg/dL, the CDC reference value [38]. No statistical difference was observed between metal biomarker levels and sex in bivariate analysis (data not shown). MnH and MnTn medians (ranges) were 0.74 (0.16–8.79) µg/g and 0.85 (0.15–13.30) µg/g, respectively, and no result was below the method’s LOD.

Child and maternal IQ scores are summarized in Table 1. Spearman correlation coefficients are presented in Table 3, which shows that children’s IQ scores are positively correlated with maternal IQ (Sp rho 0.413, *p* < 0.001), inversely correlated with their age (rho = −0.287, *p* < 0.001), with PbB levels (rho = −0.264, *p* < 0.001) and also with MnH levels (rho = −01.69, *p* = 0.017). No significant correlations were observed with the following variables: Sex (rho = 0.008), nutritional status H/A z-score (rho = 0.038), MnTn (rho = −0.018), and hemoglobin levels (rho = 0.111). On the other hand, an interesting correlation was observed between maternal IQ scores and child MnH levels (rho = −0.216, *p* = 0.006). PbB was positively correlated with child age (rho = 0.220, *p* = 0.0010), however it was inversely correlated with child chronic nutritional status H/A z-score (rho = −0.164, *p* = 0.019), and with Hb levels (rho = −0.178, *p* = 0.008).

### 3.3. MLR Models for Children’s Total IQ Scores

Data in Table 4 summarize the multiple linear regression models for children’s total IQ scores as dependent variable. Model A presents the non-standardized β-coefficients for the covariates without adjusting for maternal IQ. Negative association was observed for children’s age (β = −0.19, *p* < 0.001) LogPbB (β = −9.91, *p* = 0.001) and LogMnH levels (β = −8.09, *p* = 0.011). However, an intriguing positive association was observed for LogMnTn (β = 6.96, *p* = 0.019).

**Figure 2:**
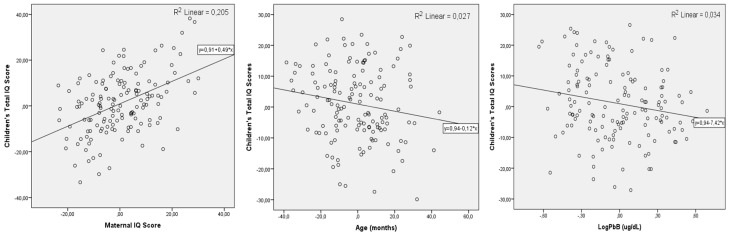
Scattered residual plots of the significant variables modeled explaining the variations in child IQ: parenteral IQ, children’s age and PbB levels.

The model B summarizes these coefficients after maternal IQ scores were entered. It shows that LogPbB β-coefficients are practically unaffected (β = −8.61, *p* = 0.004). Mn biomarkers are no longer significantly associated with child IQ. The model’s statistics are improved significantly; the correlation coefficient (*r*^2^) changes from 0.167 to 0.316, and the *F*-value changed from 6.00 to 13.88. Figure 2 presents the residual plots of children’s IQ scores for each of the significant covariates. Maternal IQ responds for 20.1% of the variance of the child IQ, followed by age (2.7%) and LogPbB levels for 3.4% of the variance.

**Figure 3:**
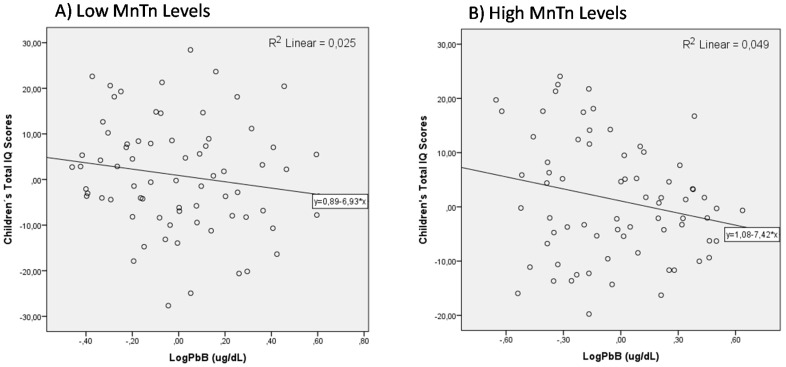
The effect modification of the low Mn (**A**) versus high Mn (**B**) exposure on the association of PbB levels on child IQ.

The effect of Mn co-exposure on the association of PbB on children’s intellectual performance was evaluated initially as an interaction. The coefficients in Model C show that the interaction terms are not significantly associated with children’s total IQ scores. However, when we tested effect modification by dichotomizing children’s Mn exposure measured by MnTn levels (Low MnTn vs. High MnTn, median 0.84 µg/g), we observed a significant change on the association of children’s PbB and their IQ scores. Data from Model D show that for children with low MnTn, the non-standardized β-coefficient for LogPbB (β = −6.80) is no longer significant (*p* = 0.172). The model’s *F*-value is decreased to 5.90 and the correlation coefficient is decreased to *r*^2^ = 0.256. On the other hand, when we run the model for children with high MnTn (Model E), the non-standardized β-coefficient for LogPbB is −8.70 (*p* = 0.036). The model’s *F*-value is 12.38 and explains 38.1% of the variance with the same number of participants (*n* = 74). The scatter plots of adjusted relation between IQ and the concurrent blood Pb concentrations in the high and low level Mn groups are shown in Figure 3a,b, respectively.

## 4. Discussion

This study provides further evidence that low lead exposure is significantly associated with cognitive impairment in school-aged children. Moreover, it was possible to observe that among children with excessive environmental exposure to manganese, the strength of the association between PbB levels and children’s IQ scores increased by approximately 58% when compared to children less exposed to Mn regardless of maternal IQ, child’s sex, and age.

The median value of PbB observed in this study is quite low (1.15 μg/dL) and 98.3% of the children had levels below 5.0 μg/dL, the CDC reference value. This median is very similar to that we reported in a previous study with children aged 1 to 11 years in the same region of this current study in 2012: 1.65 μg/dL (0.2–6.71) [13]. Similar to that study, we observed no difference in PbB levels between the sexes, and a weak positive correlation of PbB levels with age: rho = 0.256 and rho = 0.229 (*p* < 0.001) in the previous and current study, respectively. PbB levels reflect children’s exposure patterns. Olivero-Verbel et al. [39], in a study in Colombia, detected an inverse correlation (rho = −0.191, *p* = 0.009) of PbB and child age. On the other hand, a study of Moroccan children showed, an increase of PbB with age, which the authors associated with new diet and more mobility of older children [40]. In the present study, the positive correlation with age may occur because older children tend to play more outdoors, increasing their contact with soil particles with greater respiratory demand, and beside that Pb is a cumulative toxic heavy metal that is deposited in bones. Ji et al. [41] observed that workers in occupations such as construction and extractive craft workers, and installation, maintenance, and repair craft had the highest bone lead concentrations. In our cohort, only 10% of the children’s fathers had occupational activities related to the ones reported above, which could have some role on children’s Pb exposure.

We also detected a weak negative, but significant, correlation of PbB and hemoglobin concentration in those children. This could be a consequence of Pb interfering with the biosynthesis of Heme group [8]. Similarly, an inverse correlation of PbB levels and the H/A z-score, chronic malnutrition index, which may result from greater gastro-intestinal absorption of Pb in the diet and deposited dust on food. An inverse relationship has been observed between dietary calcium intake and PbB, since gastrointestinal absorption of lead is influenced by dietary and nutritional calcium and iron status [8].

We have previously published that Mn dust deposition rates in the elementary schools in the study area increase logarithmically with decreasing distance to the ferro-manganese alloy plant, and both biomarkers (MnH and MnTn) in children attending those schools were significantly correlated with these levels [33]. MnH levels observed are approximately one order of magnitude lower than what we observed previously in children of the same region living closer to the alloy plant [42], probably due to a different cleaning procedure using an extra step with 1N nitric acid. These levels are comparable with results reported by Lucas et al. [43] in Italian children and by Haynes et al. [44] with children living near a smelter in the US.

The neurodevelopmental effects of Pb exposure on children have been extensively studied and several researches report that there is no safe level of lead [16,31,32,33]. In this study, the average child IQ score was low, like what we observed previously with children from the same region with no difference between boys and girls in 2011 [42]. A series of RLM modeling was performed with IQ as the dependent variable and LogPbB as the main predictor. In the initial model we did not adjust for maternal IQ since these data were the most limiting. All metal biomarkers were significantly associated with the outcome. The non-standardized β-coefficients for LogPbB, LogMnH, and LogMnTn were −9.91, −8.09 and intriguingly positive 6.96, respectively, after adjusting for age, sex, and H/A z-score. These last two covariates were not significantly associated with IQ, and thus were not further included in the models. Since it is well described in the literature, maternal IQ is one of the most important predictors for a child’s IQ. When this covariable was entered (Model B in Table 4), the statistics are improved, only LogPbB remains significantly associated with IQ (β = −8.60, *p* = 0.004). This non-standardized coefficient is very similar to that observed in other studies [16,28]. Therefore, the decrement associated with an increase in PbB concentration from 0.5 μg/dL to 5.0 μg/dL was 8.6 IQ points, independent of maternal IQ and child age.

The interaction effect of Mn exposure on the association between cognitive deficit and PbB levels was assessed using multiplicative variables of metal biomarkers: (LogPbB*LogMnH) and (LogPbB*LogMnTn). No interaction between Mn levels in toenail and blood lead. Although an effect modification of Mn exposure on the association of PbB and IQ deficit was observed. In children, with MnTn below the median, the association between LogPbB and IQ was not significant. On the other hand, in children who had MnTn levels above the median, the association of LogPbB and IQ was significant. This reflects an increase of about 28% in the association when Mn exposure is higher. Lately, several other studies have observed the interaction effect of these two metals that share similar mechanism of action. For example, Claus-Henn et al. [29] in a Mexican cohort study observed that the slopes for the estimated 12-month lead effect on 18-month mental development scores were steeper for children with high manganese than for children with mid-range manganese levels. A similar result was observed by Lin et al [32] that found that manganese and lead levels above the 75th percentile in cord blood had a significant adverse association on cognitive functions of Taiwanese babies. The authors concluded that an interaction between the Mn and Pb levels could aggravate the harmful effect. In a birth cohort study in Bangladesh, where there were multiple exposures to Pb, Mn, and arsenic, authors have observed multiple interaction effects on the neurodevelopment of children [30,31].

Finally, we observed a significant inverse correlation between maternal IQ and child MnH levels. This negative association could have two interpretations. Mothers or caregivers with better IQs may have better schooling and possibly better SES with their houses more organized and cleaner; thus, their children would be less environmentally exposed. Another, possibility is that MnH observed in children could be a proxy of their parents’ exposure to environmental Mn, which means living closer to the ferro-manganese alloy plant. Thus, the inverse association could be interpreted as the effect of Mn exposure on parents’ intellectual function itself. Such association of child biomarkers level with maternal intellectual performance was also observed in our previous study in 2011 [42].

This study had many limitations, one of which was that we could not measure Pb and Mn in air (PM_2.5_) to estimate air exposure as previously planned. Another limitation was that we failed in collecting the whole set of data for the population enrolled in the study protocol, especially maternal IQ, which limited our data analysis and reduced the statistical power. The design character itself, since it is a cross-sectional study, could not address temporality, that is, the exposure should precede the effects.

## 5. Conclusions

Blood Pb in children was low with only 1.8% above the CDC reference value of 5 µg/dL. Despite these low PbB levels, we found that child IQ drops by 8.6 points when PbB increases from 0.5 to 5.0 µg/dL, after adjusting for maternal intellectual function, age, and Mn biomarker levels. We also observed an effect modification of Mn exposure. In children classified as less exposed to Mn, the PbB association with IQ was not significant (β = −6.80, *p* = 0.172); however, in those more exposed to Mn, the strength of the association increased by 28% and was significantly associated (β = −8.70, *p* = 0.036), after adjusting by the same covariables. The findings of this study add to the growing evidence that there is no safe level for lead and that co-exposure to Mn may increase its neurotoxic effect.

## Figures and Tables

**Table 1:** Distribution of socio-demographic, characteristics, lifestyle and IQ of the children and parents or caregivers (*n* = 226).

Characteristics	*n*	%/Mean (Min-Max)
Gender		
Boys	111	49.1%
Girls	115	50.9%
Age (months)	224	116.8 (84.0–156.9)
BMI (kg/m^2^)	211	17.62 (12.70–44.75)
H/A z-score	212	0.065 (−3.03–3.93)
Anemia	186	10 (5.5%)
Main caregiver		
Mother	148	83.6%
Father	11	6.2%
Other	18	10.2%
Gestational period in the region	216	170 (75.2%)
Smoker at home	215	39 (18.1%)
Maternal schooling		
Completed high school	215	50 (24.0%)
Less than high school	208	158 (76.0%)
Brazilian socioeconomic classification		
B	5	2.4%
C	93	41.2%
D/E	155	52.8%
Children’s IQ	217	75.5 (42–116)
Maternal IQ	174	68.9 (43–108)

**Table 2:** Lead and manganese biomarker levels in school-aged children.

Biomarker	*n*	Median	Min.–Max.	Mean	SD	P_25_–P_75_
PbB (µg/dL)	219	1.15	0.2–15.6	1.64	1.56	0.6–2.1
MnH (µg/g)	208	0.74	0.16–8.79	1.14	1.19	0.50–1.32
MnTn (µg/g)	198	0.84	0.15–13.30	1.43	1.75	0.51–1.77

**Table 3:** Spearman’s correlation matrix. Data are Spearman correlation coefficients (rho) and *p*-values.

	Children’s IQ	Maternal IQ	Sex	Age (months)	H/A z-Score	PbB (µg/dL)	MnH (µg/g)	MnTn (µg/g)	Hb (g/dL)
**Children’s IQ**	1.000	0.413 **	0.008	−0.287 **	0.038	−0.264 **	−0.169 *	−0.018	0.110
		0.000	0.906	0.000	0.591	0.000	0.017	0.807	0.111
**Maternal IQ**		1.000	0.098	−0.117	0.044	−0.070	−0.216 **	0.016	0.126
			0.198	0.128	0.567	0.367	0.006	0.846	0.102
**Sex**			1.000	0.007	−0.021	0.088	0.099	0.122	0.020
				0.917	0.760	0.193	0.153	0.087	0.766
**Age (months)**				1.000	−0.099	0.229 **	0.005	0.133	−0.013
					0.150	0.001	0.946	0.063	0.851
**H/A z-score**					1.000	−0.164 *	−0.050	0.115	−0.029
						0.019	0.490	0.117	0.677
**PbB**						1.000	0.185 **	0.244 **	−0.178 **
							0.008	0.001	0.008
**MnH**							1.000	0.394 **	−0.133
								0.000	0.058
**MnTn**								1.000	−0.164 *
									0.022
**Hb (g/dL)**									1.000

** Correlation significant at 0.01 (bilateral) and * Correlation significant at 0.05 (bilateral).

**Table 4:** Summary of the multiple linear regression models with children’s total IQ scores as a dependent variable.

**Model A: Unadjusted for Parent. IQ**	**Beta**	**Std. Error**	**T-Stat**	***p*-value**
Constant	98.118	6.334	15.492	<0.001
Age (months)	−0.195	0.053	−3.659	<0.001
Sex	0.958	1.910	0.501	0.617
H/A z-score	−0.662	0.901	−0.735	0.464
LogPbB	−9.915	3.041	−3.261	0.001
LogMnH	−8.093	3.041	−2.576	0.011
LogMnTn	6.961	2.935	2.372	0.019
Models’ statistics: *n* = 186, *r*^2^ = 0.167, F = 6.00, *p* < 0.001				
**Model B: Adjusted for Maternal IQ**	**Beta**	**Std. Error**	**T-Stat**	***p*** **-value**
Constant	61.404	8.834	6.951	<0.001
Age (months)	−0.140	0.053	−2.631	0.009
LogPbB	−8.609	2.965	−2.904	0.004
LogMnH	−2.589	3.234	−0.801	0.425
LogMnTn	4.372	2.854	1.523	0.128
ParentMaternal IQ	0.452	0.078	5.799	<0.001
Models’ statistics: *n* = 155, *r*^2^ = 0.316, F = 13.88, *p* < 0.001				
**Model C: Interactions**	**Beta**	**Std. Error**	**T-Stat**	***p*** **-value**
Constant	59.016	8.899	6.632	<0.001
Age (months)	−0.131	0.054	−2.420	0.017
LogPbB	−9.008	3.121	−2.288	0.005
LogMnH	−0.279	3.121	−0.089	0.929
Maternal IQ	0.473	0.079	5.961	<0.001
LogPbB*LogMnH	−14.317	10.868	−1.317	0.190
LogPbB*LogMnTn	6.412	8.657	0.741	0.460
Models’ statistics: *n* = 149, *r*^2^ = 0.286, F = 10.93, *p* < 0.001				
**Model D: Effect modif. Low MnTn**	**Beta**	**Std. Error**	**T-Stat**	***p-*** **value**
Constant	54.282	12.450	4.360	<0.001
Age (months)	−0.050	0.083	−0.610	0.544
LogPbB	−6.801	4.927	−1.380	0.172
LogMnH	−5.329	4.675	−1.140	0.258
Maternal IQ	0.383	0.106	3.632	0.001
Models’ statistics: *n* = 73, *r*^2^ = 0.256, F = 5.90, *p* < 0.001				
**Model E: Effect modif. High MnTn**	**Beta**	**Std. Error**	**T-Stat**	***p*** **-value**
Constant	55.445	13.843	4.005	<0.001
Age (months)	−0.170	0.073	−2.336	0.022
LogPbB	−8.699	4.064	−2.140	0.036
LogMnH	3.078	4.277	0.720	0.4474
Maternal IQ	0.598	0.124	4.812	<0.001
Models’ statistics: *n* = 74, *r*^2^ = 0.381, F = 12.38, *p* < 0.001.				

## References

[1] UNEP (United Nations Environment Program) 2012 Partnership for Clean Fuels and Vehicles. http://staging.unep.org/transport/pcfv/PDF/Brochurelead.pdf.

[2] Trasande L., Liu Y. (2011). Reducing the staggering costs of environmental disease in children, estimated at $76.6 billion in 2008. Health Aff..

[3] Gould E. (2009). Childhood lead poisoning: Conservative estimates of the social and economic benefits of lead hazard control. Environ. Health Perspect..

[4] CDC (Centers for Disease Control and Prevention) Interpreting and Managing Blood Lead Levels <10 ug/dl in Children and Reducing Childhood Exposures to Lead: Recommendations of CDC’s Advisory Committee on Childhood Lead Poisoning Prevention. http://www.cdc.gov/mmwr/PDF/rr/rr5608.pdf.

[5] Obeng-Gyasi E. (2018). Lead Exposure and Oxidative Stress—A Life Course Approach in US Adults. Toxics.

[6] Ide L.S., Parker D.L. (2005). Hazardous Child Labor: Lead and Neurocognitive Development. Public Health Rep..

[7] Lo Y.C., Dooyema C.A., Neri A., Durant J., Jefferies T., Medina-Marino A., de Ravello L., Thoroughman D., Davis L., Dankoli R.S. (2012). Childhood lead poisoning associated with goldore processing: A village-level investigation-Zamfara State, Nigeria. Environ Health Perspect..

[8] ATSDR—Agency for Toxic Substances and Disease Registry (2010). Toxicological Profile for Lead.

[9] World Health Organization (WHO) (2011). Summary of Principles for Evaluating Health Risks in Children Associated with Exposure to Chemicals.

[10] Lidsky T.I., Schneider J.S. (2003). Lead neurotoxicity in children: Basic mechanisms and clinical correlates. Brain.

[11] World Health Organization Childhood Lead Poisoning. http://www.who.int/ceh/publications/leadguidance.pdf.

[12] AAP Council on Environmental Health (2016). Prevention of Childhood Lead Toxicity. Pediatrics.

[13] Menezes-Filho J.A., Viana G.F.S., Paes C.R. (2012). Determinants of lead exposure in children on the outskirts of Salvador, Brazil. Environ. Monit. Assess..

[14] Needleman H.L., Riess J.A., Tobin M.J., Biesecker G.E., Greenhouse J.B. (1996). Bone lead levels and delinquent behavior. JAMA.

[15] Nigg J.T., Knottnerus G.M., Martel M.M., Nikolas M., Cavanagh K., Karmaus W., Rappley M.D. (2008). Low blood lead levels associated with clinically diagnosed attention deficit/hyperactivity disorder and mediated by weak cognitive control. Biol. Psychiatry.

[16] Lanphear B.P., Hornung R., Khoury J., Yolton K., Baghurst P., Bellinger D.C., Canfield R.L., Dietrich K.N., Bornschein R., Greene T. (2005). Low-level environmental lead exposure and children’s intellectual function: An international pooled analysis. Environ. Health Perspect..

[17] Froehlich T.E., Lanphear B.P., Auinger P., Hornung R., Epstein J.N., Braun J., Kahn R.S. (2009). The association of tobacco and lead exposure with attention-deficit/hyperactivity disorder. Pediatrics.

[18] Johnson M.H. (2003). Development of human brain functions. Biol. Psychiatry.

[19] Casey B.J., Tottenham N., Liston C., Durston S. (2005). Imaging the developing brain: What have we learned about cognitive development?. Trends Cogn. Sci..

[20] Suszkiw J.B. (2004). Presynaptic Disruption of Transmitter Release by Lead. Neurotoxicology.

[21] Karri V., Schuhmacher M., Kumar V. (2016). Heavy metals (Pb, Cd, As and MeHg) as risk factors for cognitive dysfunction: A general review of metal mixture mechanism in brain. Environ. Toxicol. Pharmacol..

[22] Guilarte T.R., McGlothan J.L., Degaonkar M., Chen M.K., Barker P.B., Syversen T., Schneider J.S. (2006). Evidence for cortical dysfunction and widespread manganese accumulation in the nonhuman primate brain following chronic manganese exposure: A 1HMRS and MRI study. Toxicol. Sci..

[23] Aschner M., Guilarte T.R., Zheng W. (2007). Manganese: Recent advances in understanding its transport and neurotoxicity. Toxicol. Appl. Pharmacol..

[24] Guilarte T.R., Costa L.G., Aschner M. (2015). A decade of studies on manganese neurotoxicity in non-human primates: Novel findings and future directions. Manganese in Health and Disease.

[25] Chen P., Chakraborty S., Mukhopadhyay S., Lee E., Paoliello M.M., Bowman A.B., Aschner M. (2015). Manganese homeostasis in the nervous system. J. Neurochem..

[26] Bouabid S., Tinakoua A., Lakhdar’Ghazal N., Benazzouz A. (2016). Manganese neurotoxicity: Behavioral disorders associated with dysfunctions in the basal ganglia and neurochemical transmission. J. Neurochem..

[27] Guilarte T.R. (2013). Manganese neurotoxicity: New perspectives from behavioral, neuroimaging, and neuropathological studies in humans and non-human primates. Front. Aging Neurosci..

[28] Lucchini R.G., Zoni S., Guazzetti S., Bontempi E., Micheletti S., Broberg K., Parrinello G., Smith D.R. (2012). Inverse association of intellectual function with very low blood lead but not with manganese exposure in Italian adolescents. Environ. Res..

[29] Claus Henn B., Schnaas L., Ettinger A.S., Schwartz J., Lamadrid-Figueroa H., Hernandez-Avila M., Amarasiriwardena C., Hu H., Bellinger D.C., Wright R.O. (2012). Associations of early childhood manganese and lead coexposure with neurodevelopment. Environ. Health Perspect..

[30] Rodrigues E.G., Bellinger D.C., Valeri L., Hasan M.O.S.I., Quamruzzaman Q., Golam M., Kile M.K., Christiani D.C., Wright R.O., Mazumdar M. (2016). Neurodevelopmental outcomes among 2- to 3-year-old children in Bangladesh with elevated blood lead and exposure to arsenic and manganese in drinking water. Environ. Health.

[31] Valeri L., Mazumdar M.M., Bobb J.F., Claus Henn B., Rodrigues E., Sharif O.I.A., Kile M.L., Quamruzzaman Q., Afroz S., Golam M. (2017). The Joint Effect of Prenatal Exposure to Metal Mixtures on Neurodevelopmental Outcomes at 20–40 Months of Age: Evidence from Rural Bangladesh. Environ. Health Perspect..

[32] Lin C.C., Chen Y.C., Su F.C., Lin C.M., Liao H.F., Hwang Y.H., Hsieh W.S., Jeng S.F., Su Y.N., Chen P.C. (2013). In utero exposure to environmental lead and manganese and neurodevelopment at 2 years of age. Environ. Res..

[33] Rodrigues J.L.G., Bandeira M.J., Araújo C.F.S., Santos N.R., Anjos A.L.S., Koin N.L., Pereira L.C., Oliveira S.S.P., Mergler D., Menezes-Filho J.A. (2017). Manganese and lead levels in settled dust in elementary schools are correlated with biomarkers of exposure in school-aged children. Environ. Pollut..

[34] Menezes-Filho J.A., de Souza K.O.F., Rodrigues J.L.G., dos Santos N.R., de Jesus Bandeira M., Koin N.G., Mergler D. (2016). Manganese and lead in dust fall accumulation in elementary schools near a ferromanganese alloy plant. Environ. Res..

[35] Associação Brasileira de Empresas de Pesquisa—ABEP (2011). Critério de Classificação Econômica Brasil.

[36] World Health Origination WHO (2009). AnthroPlus for Personal Computers Manual: Software for Assessing Growth of the World’s Children and Adolescents.

[37] Wechsler D. (2002). Escala de Inteligência Wechsler para Crianças, (WISC-III): Manual; Adaptação e Padronização de uma Amostra Brasileira.

[38] CDC (Centers for Disease Control and Prevention) Blood Lead Levels in Children. http://www.cdc.gov/nceh/lead/ACCLPP/Lead_Levels_in_Children_Fact_Sheet.pdf.

[39] Olivero-Verbel J., Duarte D., Echenique M., Guette J., Johnson-Restrepo B., Parsons P.J. (2017). Blood lead levels in children aged 5–9 years living in Cartagena, Colombia. Sci. Total Environ..

[40] Zaida F., Chadrame S., Sedki A., Lekouch N., Bureau F., Arhan P., Bougle D. (2007). Lead and aluminium levels in infants’ hair, diet, and the local environment in the Moroccan city of Marrakech. Sci. Total Environ..

[41] Ji J.S., Schwartz J.S., Sparrow D., Hu H., Weisskopf M.G. (2014). Occupational Determinants of Cumulative Lead Exposure: Analysis of Bone Lead among Men in the VA Normative Aging Study. J. Occup. Environ. Med..

[42] Menezes-Filho J.A., Novaes C.O., Moreira J.C., Sarcinelli P.N., Mergler D. (2011). Elevated manganese and cognitive performance in school-aged children and their mothers. Environ. Res..

[43] Lucas E.L., Bertrand P., Guazzetti S., Donna F., Peli M., Jursa T.P., Lucchini R., Smith D.R. (2015). Impact of ferromanganese alloy plants on household dust manganese levels: Implications for childhood exposure. Environ. Res..

[44] Haynes E.N., Sucharew H., Hilbert T.J., Kuhnell P., Spencer A., Newman N.C., Burns R., Wright R., Parsons P.J., Dietrich K.N. (2018). Impact of air manganese on child neurodevelopment in East Liverpool, Ohio. Neurotoxicology.

